# Process optimization for the rapid conversion of calcite into hydroxyapatite microspheres for chromatographic applications

**DOI:** 10.1038/s41598-022-16579-4

**Published:** 2022-07-16

**Authors:** Anbuthangam Ashokan, T. S. Sampath Kumar, Guhan Jayaraman

**Affiliations:** 1 Department of Metallurgical and Materials Engineering, Indian Institute of Technology Madras, Chennai, 600036 India; 2 Department of Biotechnology, Indian Institute of Technology Madras, Chennai, 600036 India

**Keywords:** Biomaterials, Biomedical engineering

## Abstract

Microsphere hydroxyapatite (HAp) is widely used in various biomedical and chromatographic applications. The work described in this manuscript focuses on a dissolution precipitation method for production of HAp microspheres. This method overcomes certain drawbacks of conventional preparation methods used for HAp preparation, which produce polydisperse particles and are time-consuming and expensive. In the present work, the calcium carbonate (calcite) particles were directly and rapidly converted into HAp microspheres using an inexpensive dissolution precipitation method. The effects of the reaction temperature, time, and mechanical stirring rates were studied, and the reaction parameters were optimized. As confirmed by the XRD studies, the higher reaction temperature and time promote complete HAp conversion, while calcite residues were observed for lower temperatures and times. SEM images show the influence of reaction parameters on the surface microstructure of the microspheres produced. It was observed that the HAp microspheres undergo disintegration at a higher stirring rate. The reaction parameters optimized in this work were ideal for preparing HAp microspheres. The resultant HAp particles were utilized as matrices for chromatographic separation of protein mixtures.

## Introduction

Hydroxyapatite (HAp) is a multi-purpose inorganic material with applications in the field of medicine, wastewater treatment, sensor, catalysis, and chromatography^1,2^. Among the various morphologies of HAp, the microsphere morphology is preferred because of its unique physical characteristics such as large surface area, better protein adsorption capacity, and superior flowability^3,4^. Therefore, several fabrication methods have been proposed for microsphere preparation. HAp (Ca_5_(PO_4_)_3_OH) is a well studied calcium phosphate (CaP) mineral with a hexagonal crystalline structure. However, the production of HAp microspheres with uniform properties remains a challenge due to the notable dissimilarity in crystallographic structure, chemical composition, phase stability, dissolution behavior, crystallization thermodynamics, and kinetics of the different forms of CaPs^5,6^.

Various methods have been developed to prepare HAp, such as biomimetic technique, solid–solid reaction, spray-drying, sol–gel, wet precipitation, emulsion, microwave-assisted synthesis, hydrothermal and template-assisted synthesis, etc.^1,4^. The precipitation process with aqueous orthophosphate and calcium sources appears to be the most common technique to prepare HAp particles. In this route, water-soluble calcium sources such as calcium chloride, calcium acetate, or calcium nitrate are preferred because of the resultant homogeneity of the reaction medium. Subsequently, highly pure HAp can be obtained for better quality materials in biomedical and chromatographic applications. However, these soluble calcium precursors present some disadvantages regarding their relative expense compared to the less water-soluble calcium precursors like calcium carbonate or calcium hydroxide and the labor-intensive processes involved during HAp formation. They also need to be treated to eliminate waste contaminants from counterions^7,8^. This work has focused on relatively less expensive materials such as calcium carbonate for HAp synthesis. In environmental and purification applications, such inexpensive HAp materials are also preferred as sorbents.

Calcite naturally exists in many biological wastes such as mussel shells, eggshells, kina shells, oyster shells, snail shells, etc.^9–11^. Utilizing inexpensive calcite with reduced energy consumption protocols for HAp preparation will be a valuable research challenge^4,10^. Most methods reported in literature do not directly utilize calcium carbonate for HAp synthesis. The difficulty in directly utilizing calcium carbonate is that it has a long reaction time and cannot be wholly decomposed into HAp at room temperature. Calcite in waste materials are usually converted into calcium oxide by thermal treatment or into water-soluble calcium salt by acid solubilization route^2^. The hydrothermal method is a highly reported technique for converting calcium carbonate into HAp^3^. However, in the hydrothermally-treated mixtures of calcium carbonate and (NH_4_)_2_HPO_4_, it is observed that the conversion is slow and incomplete for a ball-and-block structure of calcium carbonate when compared to a rod-shaped carbonate^7,12^. Highly crystalline particles with uniform morphology can be synthesized by the hydrothermal method but the outcome is in small scale and associated with high costs. Moreover the hydrothermal method is time-consuming and involves complicated steps^13^. The literature suggests that the calcium carbonate conversion reaction depends on various parameters like liquid–solid (L/S) ratio, precursor shape, reaction time, and temperature^1^. There is not much literature on the effect of stirring conditions on calcite into HAp conversion. Even though a lot of information has been obtained regarding converting calcium carbonate to HAp, it is essential to optimize the parameters affecting the quantitative conversion of calcite into HAp. The current work focuses on the reaction parameter optimization for a single-step process for the direct and complete transformation of calcite to HAp. Different process parameters which influence conversion were examined, including reaction time, temperature, and stirring rate^12^.

Finally, the HAp microspheres prepared by this method were used as matrices for chromatographic separation of protein mixtures.

## Materials and methods

### Chemicals

Bovine serum albumin and lysozyme were purchased from HiMedia Laboratories Pvt. Ltd (India). Calcium chloride (CaCl_2_), ammonium phosphate dibasic ((NH_4_)_2_HPO_4_), sodium hydroxide (NaOH), anhydrous sodium carbonate (Na_2_CO_3_), and ammonia solution were obtained from Rankem Avantor Performance Materials India Limited (Maharastra, India). Sodium phosphate monobasic (NaH_2_PO_4_) and sodium phosphate dibasic (Na_2_HPO_4_) were obtained from Merck Life Science Private Limited (Mumbai, India). All chemicals were of analytical grade.

### Preparation of calcium carbonates (calcite)

Micron-sized calcium carbonate (calcite) samples were prepared by the chemical precipitation method at ambient temperature. Equal concentrations of calcium chloride and sodium carbonate stock solutions were prepared separately. Then the sodium carbonate stock solution was quickly added into the calcium chloride solution with intense stirring for 30 min at 500 rpm. The resultant white precipitates were filtered, washed with distilled waterand dried at room temperature.

### Conversion of calcite into HAp microparticle

The micron-size calcium carbonate was used as a calcium precursor for the HAp preparation via dissolution precipitation reaction. The calcium carbonate was added into the diammonium hydrogen phosphate solution, keeping the Ca/P ratio as 1.67, and maintaining the pH at 10. After the reaction was completed, the samples were filtered and dried in a hot air oven at 100 °C. The parameters affecting conversion—temperature, time, and stirring rates, were optimized for the resultant mixture by keeping the pH 10; since alkaline pH is a favorable environment for the HAp formation, we kept it constant throughout the experiments. The values of these parameters are summarized in Table 1 with the sample code conveying the information of synthesis conditions.

**Table 1:** Reaction variables used in the synthesis of HAp.

Samples	Stirring rate (rpm)	Reaction time (h)	Reaction temperature (°C)
S1	w/o	2	160
S2	100	2	160
S3	300	2	160
S4	500	2	160
S5	300	0.5	160
S6	300	1	160
S7	300	1.5	160
S8	300	2	160
S9	300	2	RT (room temperature)
S10	300	2	50
S11	300	2	80
S12	300	2	160

### Materials characterization

The crystalline phase purity of the products was studied with X-ray Diffractometer (XRD, Bruker D8 Discover, USA) using Cu Kα radiation (λ = 1.54 Å, with a scanning rate of 1 step/s and step size of 0.1 degrees/step). Fourier Transform Infra-Red (FTIR) spectra were measured at room temperature in the range of wavenumbers 4000–550 cm^−1^ using attenuated total reflectance (ATR) mode in FTIR (Perkin Elmer Spectrum Two FTIR spectrometer, Germany) to evaluate the functional group present in the material. The materials elemental analysis and morphology change were observed using a Scanning electron microscope with a field-emission gun (Quanta 400 FEG, SEM, USA) attached with energy dispersive X-ray spectroscopy (EDS). The surface areas and nitrogen adsorption–desorption isotherms were measured with IC-BET Micromeritics ASAP 2020 instrument at − 195.640 °C. The thermal behavior of the samples was studied by thermo-gravimetric analysis (SDTQ600-TA Instruments) in the range of 25–1000 °C in N_2_ atmosphere using alumina as reference material. Laser diffraction particle size analyzer (Microtrac S3500, USA) was used to find the particle size and distribution of HAp. Distilled water was chosen as a dispersant for the particle size measurement, and the results were given as an equivalent spherical diameter (ESD) in volume %.

### Chromatography column packing and protein separation

The prepared HAp microspheres were used for packing chromatographic columns for protein separation. Initially, 4 g of HAp microspheres were dispersed in equilibration buffer, sonicated for a few seconds to make the bubble-free suspension, and poured into XK 16/20 Column (Column Volume (CV) = 6 ml). The column was gradually packed by gravity settling, followed by applying pressure. The packed column was connected with AKTA Pure system and equilibrated with 5 mM sodium phosphate buffer for 10 CV.

A binary protein mixture consisting of bovine serum albumin (BSA) and lysozyme (LYZ) was dissolved in an equilibration buffer containing 5 mM PO_4_ buffer at pH 6.8 with the final concentration of 5 mg/ml was injected at a flow rate of 1 ml/min. The protein mixture was separated using isocratic elution with 90 mM PO_4_ buffer at pH 6.8.

## Results and discussion

**Figure 1:**
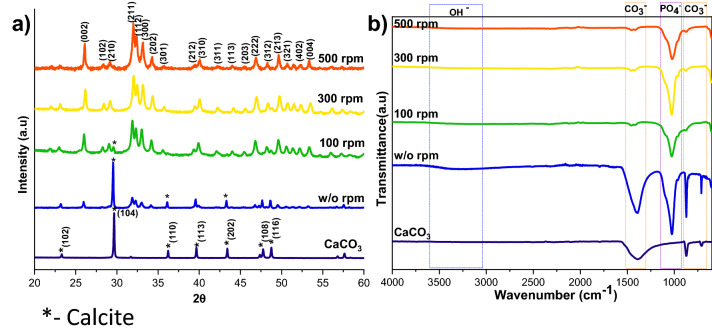
XRD (**a**), and FTIR (**b**) spectra of HAp prepared at a different stirring rate (CaCO3 JCPDS no: 05-0586 and HAp JCPDS no: 09-0432).

**Figure 2:**
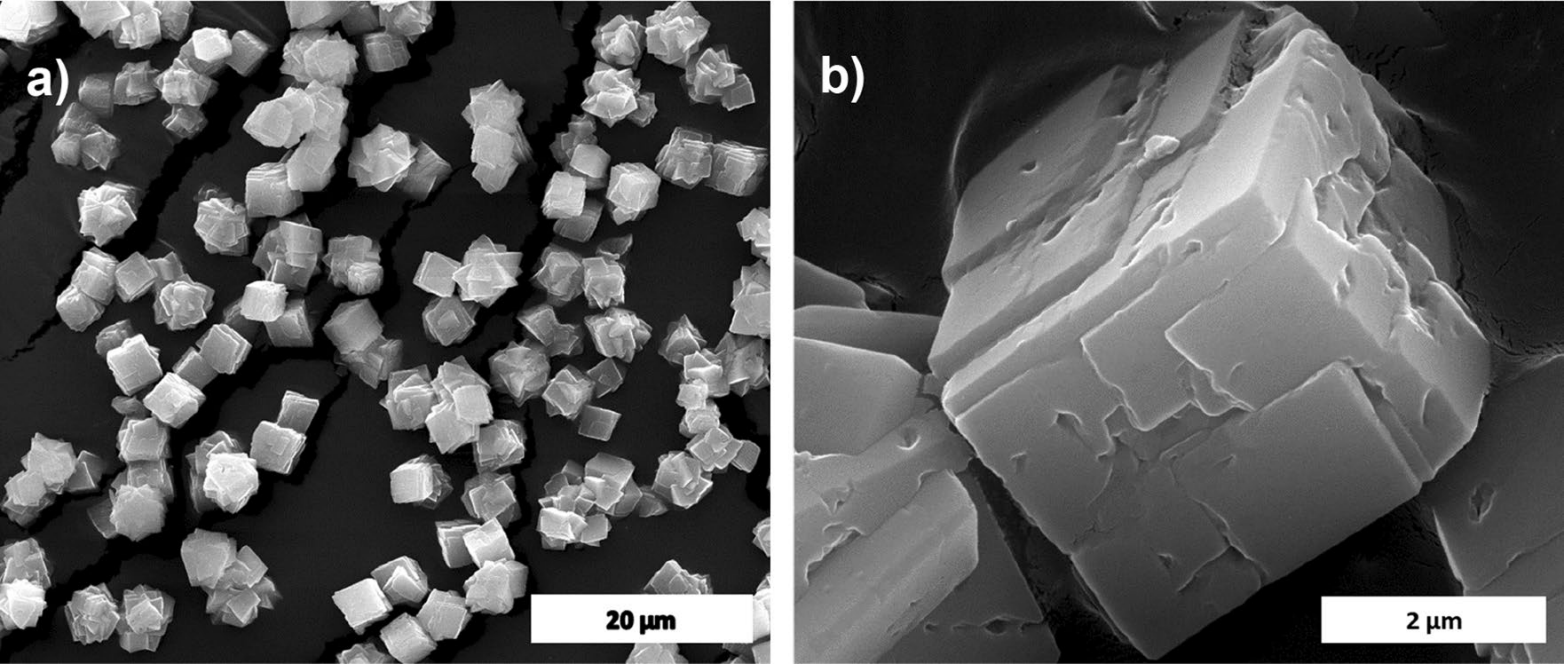
SEM image of calcite (**a**) and its magnified image (**b**).

The calcium carbonate formed in our experiment is phase-pure without any additional peak, and matched well with the standard calcite JCPDS no: 05-0586 shown in Fig. 1a. The sharp peaks indicate that the material is highly crystalline. The FTIR results shown in Fig. 1b support the XRD data; it conforms to the calcite sample formation from the characteristic vibrational bands at about 1422, 877 (v2 mode), and 712 (v4 mode) cm^−1^^12^. The appearance of the calcite phase is predominant compared to other polymorphs of calcium carbonate due to its thermodynamic stability^14,15^. The scanning electron microscope (SEM) observations (Fig. 2) showed that the sample formed is calcite crystal and compares well with results in the literature. The calcium carbonate crystal formed was a 5–10 µm cubic system (Fig. 2b), comprised of an aggregation of many tiny crystals (Fig. 2b)^16^. The SEM analysis showed that we obtained monodisperse particles (Fig. 2a), while literature reports mostly show polydisperse calcite phase particles.

### HAp formation mechanism

Calcium carbonate particles were introduced into the phosphate solution, keeping the Ca/P ratio at 1.67. The calcium carbonate solubility depends on temperature and phosphate precursor used. Ammonium phosphate salts are preferred over other salts because it is highly soluble in water and do not leave undesired cations in the reaction media, which may hinder the HAp purity. The Ca^2+^ ions were released from the calcite surface into phosphate solution, where they reacted and formed HAp via dissolution precipitation. The solubility differences between the salt precursors and the resultant product impact the respective ion exchange processes^17^. Here the solubility of calcite (log K_sp_ =  − 8.48) is higher than that of the HAp solubility (log K_sp_ =  − 118.268), so it will promote the calcite dissolution in the phosphate medium, which leads to the faster replacement of CO_3_ by PO_4_ ions^12^. Since HAp possesses lower solubility, the reprecipitation of calcium carbonate will not occur, which leads to the thermodynamically favorable HAp formation once the components are accessible. CO_2_ gas evolved from the suspension during the reaction, indicating calcium carbonate decomposition. This reaction is elaborated below (Eq. (1)).

1 $${5 \text{CaCO}}_{3} + 3 {{\left({\text{NH}}_{4}\right)}_{2}\text{HPO}}_{4}= {\text{Ca}}_{5}{\left({\text{PO}}_{4}\right)}_{3}\left(\text{OH}\right)+ 5 {\text{CO}}_{2} + 6 {\text{NH}}_{3} +4 {\text{H}}_{2}\text{O}.$$

### Parameters affecting HAp synthesis

To improve the yield of HAp synthesized from calcite, the influence of various parameters such as the reaction temperature, reaction time, and stirring rate were investigated.

#### Effect of stirring rate on calcite to HAp conversion

**Figure 3:**
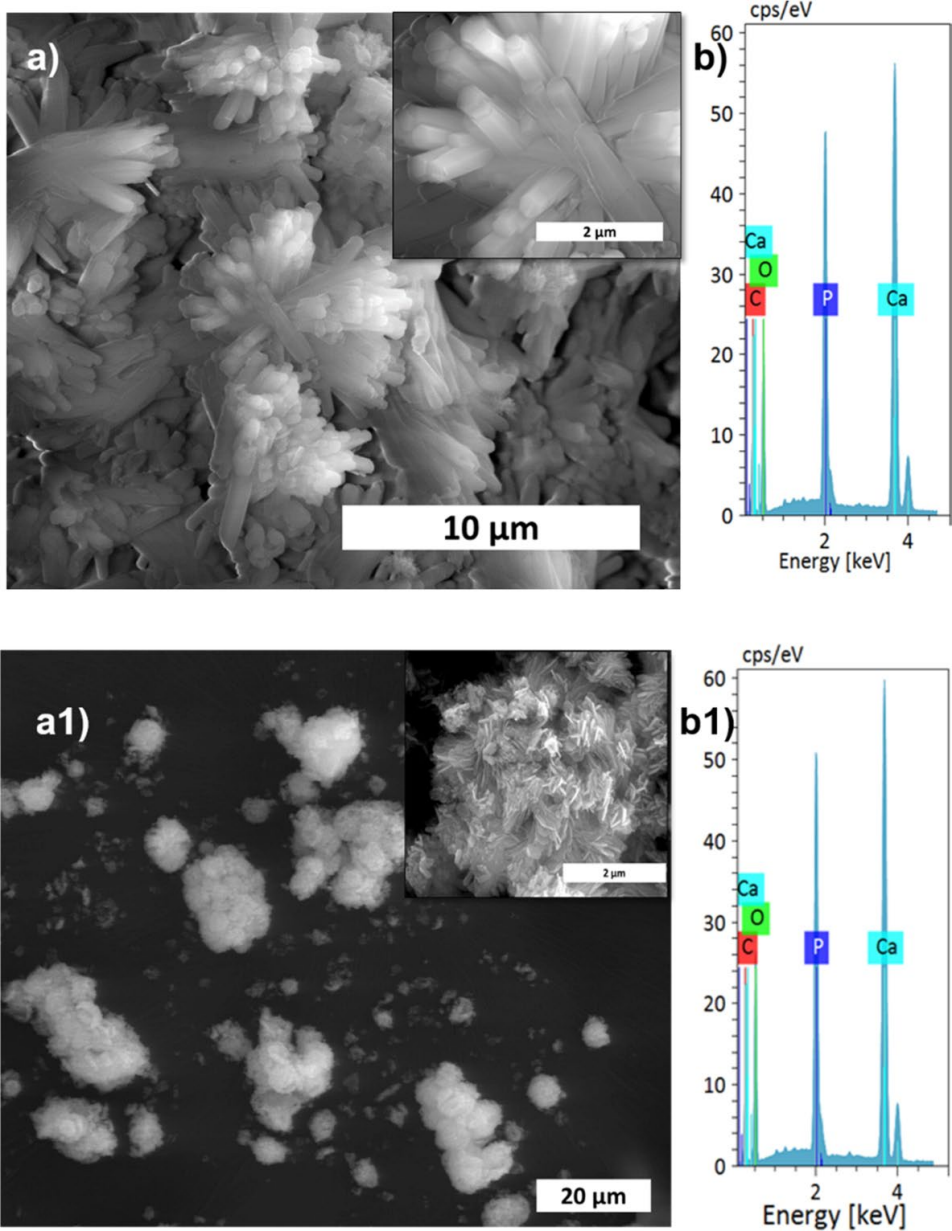
SEM analysis (**a,a1**), and its magnified images (insert), with EDS spectrum (**b,b1**) of S1, and S4 samples, respectively.

The XRD patterns of HAp, prepared at different stirring rates, are shown in Fig. 1a. The calcite phase fraction was higher than the HAp phase, confirmed by the calcite (104) plane. The intensity of the calcite plane decreased with increased stirring. The complete conversion took place when the stirring rate increased from static to 300 rpm due to the increased contact of calcite particles with the surrounding phosphate solution^1^. Further enhancement of stirring speed did not lead to additional phase change, and it showed the same crystallinity value for all the stirring conditions (Supplementary Table S1), but it affects the microstructure, as we can see from the SEM image shown in Fig. 3a1. High stirring leads to the disintegration of the particles. The non-stirred samples showed hexagonal HAp morphology that covers the calcite surface (Fig. 1a). Literature reports have observed the same hexagonal morphology for typical apatite^17^. When the stirring rate increased, the morphology changed into a plate-like structure, due to the dissolution and precipitation in all directions^18^. The EDS analysis proved that static and lower stirring samples showed minimum phosphate values indicating incomplete conversion (Fig. 3b, Supplementary Fig. S1b). The FTIR results demonstrate that samples obtained without stirring show more calcite characteristics vibration bands at 1429 cm^−1^, 876 cm^−1^, and 713 cm^−1^ (Fig. 1b). In the case of stirred samples, the characteristics peaks for the calcite phase vanished with increased stirring, and we are left with characteristic vibrations of phosphate at 1038 cm^−1^ (n3), 966 cm^−1^ (n1), and 604 cm^−1^, respectively.

#### Effect of reaction time on calcite to HAp conversion

**Figure 4:**
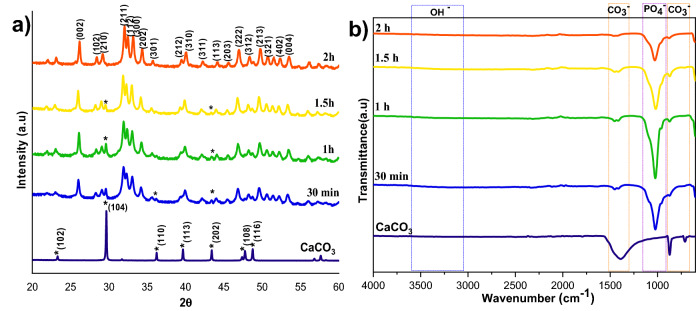
XRD (**a**) and FTIR (**b**) spectra of HAp prepared at different reaction times.

**Figure 5:**
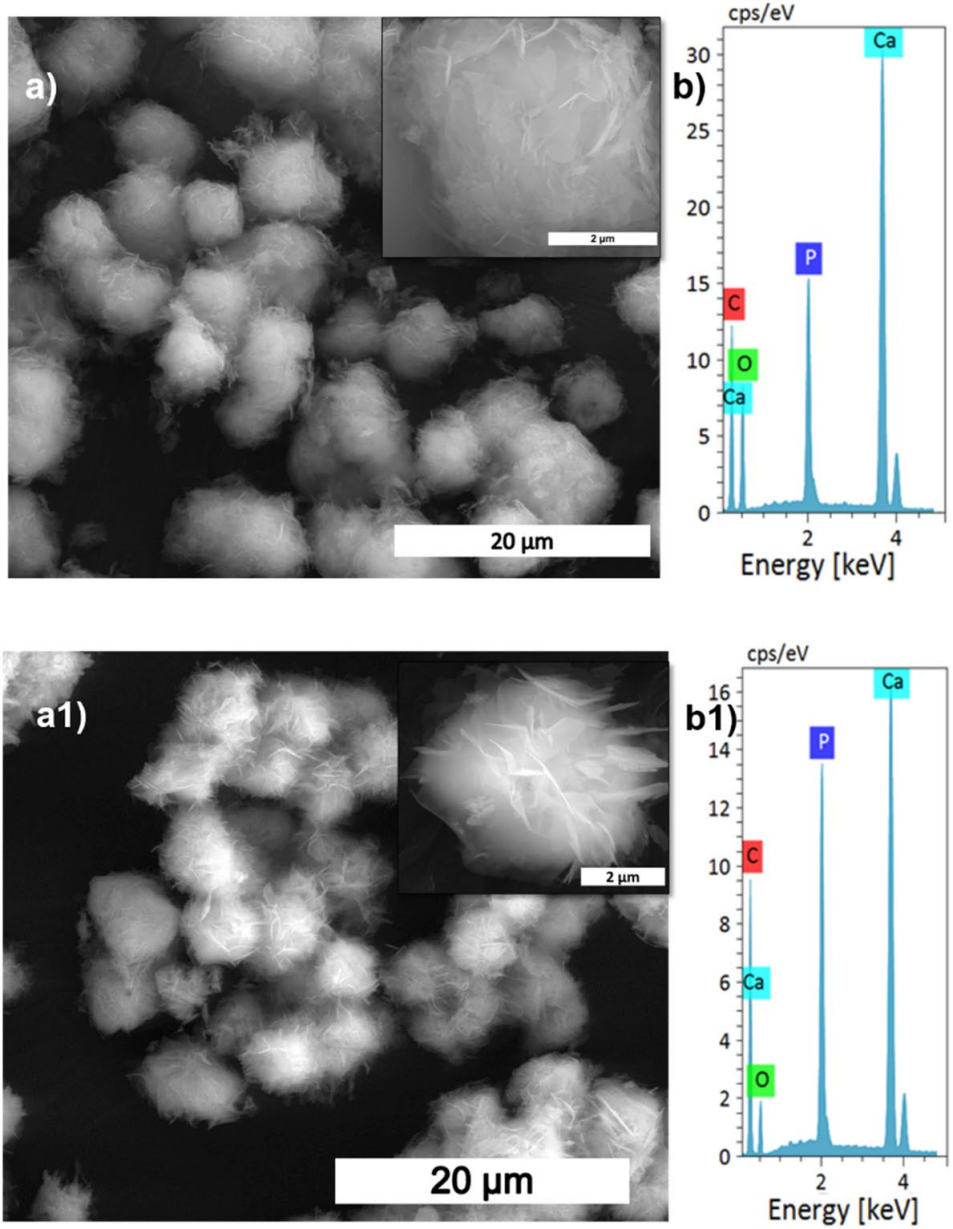
SEM analysis (**a,a1**), and its magnified images (insert), with EDS spectrum (**b,b1**) of S5, and S8 samples, respectively.

The effect of reaction time on calcite conversion into HAp XRD profile is shown in Fig. 4a. It can be seen from the characteristic peaks of calcite at 29.4° that the reaction was incomplete at 30 min, 1 h, and 1.5 h, respectively. The 2 h sample did not exhibit any trace of calcite, implying complete conversion. The results were supported by FTIR analysis; the 2 h sample showed only characteristic peaks of HAp without calcite characteristic peaks. The other samples showed typical vibration bands of carbonate in their structure. The morphological analysis was shown in Fig. 5a,a1, and Supplementary Fig. S2 confirms the samples are made of a plate-like system at an all-time point, but the size of the plate structure varied at each time point. The plate structure assembly was more clearly observed in the high reaction time samples (Fig. 5a1). Increased reaction time improved the HAp phase formation, and the Ca/P ratio was close to the ideal value of HAp. This result was supported by EDS analysis shown in Fig. 5b1 and Fig. S2b,b1. The S3 sample showed complete conversion of calcite into HAp.

#### Effect of reaction temperature on calcite to HAp conversion

**Figure 6:**
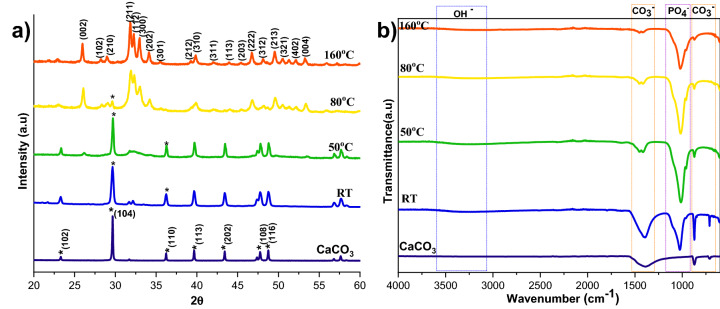
XRD (**a**) and FTIR (**b**) spectra of HAp prepared at a different reaction temperatures.

Although the calcite was wholly converted into HAp at 160 °C with a 2 h reaction, it is desirable to find the minimum temperature required to achieve the complete conversion of calcite into HAp. The XRD patterns of calcite transformation to HAp, over a 2 h reaction time, at various temperature conditions are shown in Fig. 6a. Here, the peaks were indexed according to the standard JCPDS data of calcite (05-0586) and HAp (09-0432). From XRD profiles, it can be observed that the prominent peak of the calcite phase decreased with increasing temperature; at the same time, the characteristic peak of HAp increased with rising temperature. The significant finding from the temperature analysis is that the lower temperature is insufficient to dissolve calcite completely. Complete conversion occurs at higher temperatures since the increasing temperature leads to the higher solubility of calcite^19^. The peak intensity of the HAp also increased at higher temperatures, which implies that the temperature improves the crystallinity and decreases the solubility of the HAp^20^. The crystallinity calculations in Supplementary Table S1 supported the XRD profile, proving that the rising temperature is the leading cause of the crystallinity and crystal size increase.

The FTIR spectra of HAp prepared from calcite at different temperatures are shown in Fig. 6b. The characteristic vibrational phosphate bands are observed in all the converted samples at 1038 cm^−1^ (v_3_), 966 cm^−1^ (v_1_), and 604 cm^−1^, respectively. Peaks at 3742 cm^−1^ and 669 cm^−1^ correspond to the stretching and bending vibrations of hydroxyl (OH^−^) and are considered the characteristic peaks of HAp. In addition, peaks observed at 3420 cm^−1^ are due to the tensile vibrations of adsorbed water. Peaks at 1429 cm^−1^, 876 cm^−1^, and 713 cm^−1^ are attributable to the antisymmetric expansion, out-of-plane bending, and in-plane bending of CO_3_^2−^. It indicates that part of CO_3_^2−^ may enter HAp lattice and exchange part of OH^−^ or PO_4_^2−^ to form A-type or B-type HAp. Compared with the calcite, we can see that the characteristic vibrations of phosphate improved, and the distinctive bands of CO_3_^2−^ decreased by increasing the temperature. At 160 °C the carbonate peaks almost vanished, indicating that the temperature enhances the conversion of calcite to HAp.

**Figure 7:**
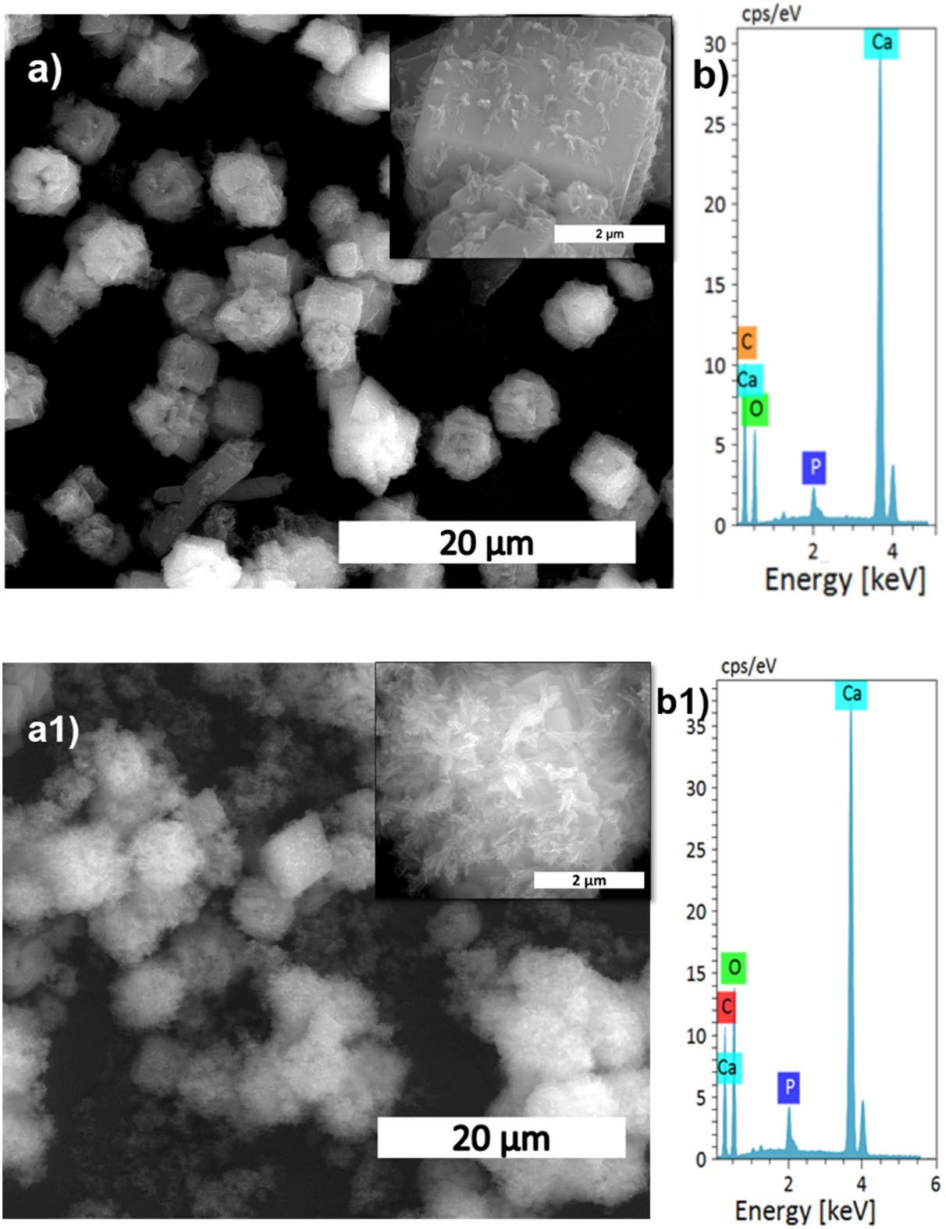
SEM analysis (**a,a1**), and its magnified images (insert), with EDS spectrum (**b,b1**) of S9, and S10 samples, respectively.

The microstructural change regarding temperature in the conversion of calcite into HAp is shown in Fig. 7a,a1, and Supplementary Fig. S3. At lower temperatures, the amount of HAp formed was significantly less, as seen in the SEM image showing the HAp coverage of the calcite surface. The HAp coverage increased when the temperature reached 50 °C compared to the room temperature sample. At 160 °C, the HAp is wholly formed and appears like a fluffy ball made of self-assembled plates (Supplementary Fig. S3a1). The conversion was further supported by EDS analysis (Fig. 7b,b1) where it shows that the Ca/P ratio decreased with temperature, indicating that the calcite was wholly converted into HAp. A higher Ca/P ratio indicates the incomplete conversion of HAp due to the replacement of phosphate by carbonate in HAp^21^.

### Determination of the remaining calcium carbonate contents

FTIR and TG analysis was performed to estimate the reaction’s progress to find the remaining calcium carbonate, the starting precursor for the HAp formation. Figures 1b, 4b, and 6b shows typical FTIR spectra in the range 600–4000 cm^−1^ for all the three different preparation conditions; where v_2_(CO_3_^2−^) and v_3_(CO_3_^2−^) IR-active vibration modes are the characteristic carbonate absorption bands evidenced around 840–900 and 1350–1550 cm^−1^ respectively^22^. The former vibration band is less intense than the latter, and it also interferes with an HPO_4_^2−^ vibrational band around 875 cm^−1^. So, the v_3_(CO_3_^2−^) vibrational contribution is preferred over the v_2_(CO_3_^2−^) contribution to calculate the remaining CO_3_^2−^ content in the HAps. Along with the carbonate bands, the characteristic features of apatitic compounds are also clearly evident on these spectra, with apatitic hydroxide bands at 3572 (OH^−^ stretching) and 632 cm^−1^ (OH liberation), as well as phosphate vibration modes v_1_(PO_4_^2−^), v_2_(PO_4_^2−^), v_3_(PO_4_^2−^), and v_4_(PO_4_^2−^) as indicated in the FTIR spectra.

The carbonate presence in HAp was evaluated from the area or intensity ratio of the 1415 cm^−1^ band relative to the v_3_(PO_4_^2−^) phosphate band is calculated (v_2_(CO_3_^2−^)/v_3_(PO_4_^2−^) area ratio), it represented as r_c/p_^22^.

wt% of carbonate from the FTIR spectra is shown below (Eq. (2)).

2 $${\text{wt}\%\text{CO}}_{3}= \left(28.62\times {r}_{c/p}\right)+0.0843,$$

where r_c/p_ is the area ratio between the v_2_(CO_3_^2−^) band (1550–1330 cm^−1^) and the v_3_(PO_4_^2−^) band (1230–900 cm^−1^)^23^. The remaining carbonate content results proved that the calcium carbonate content would reduce with increasing temperature and time; the carbonate content has almost vanished in 300 rpm samples shown in Table 2, where the Ca/P ratio was calculated from EDS spectra shown in Figs. 3b,b1, 5b,b1, 7b,b1, Supplementary Figs. S1b,b1, S2b,b1 and S3b,b1. The Ca/P ratio results confirmed that the S3 sample showed better HAp formation because of the ratio closeness to the stoichiometric value of HAp. But the, little deviation occurred due to the substitution of carbonate in the HAp structure. The remaining sample showed a higher Ca/P ratio indicating its incomplete conversion.

**Table 2:** Remaining carbonate content calculation using various methods.

Samples	FTIR method	TGA overall mass loss	TGA mass loss in CO_3_ decomposition range	Ca/P ratio
S1	28.81	23.58	17.49	2.11 ± 0.15
S2	2.11	6.48	1.89	2.22 ± 0.09
S3	1.54	3.31	1.10	1.85 ± 0.05
S4	1.63	7.0	0.92	1.98 ± 0.09
S5	2.66	8.22	2.73	2.90 ± 0.77
S6	1.83	7.24	2.64	2.14 ± 0.03
S7	1.72	6.76	2.30	2.14 ± 0.01
S8	1.54	3.31	1.10	1.85 ± 0.05
S9	28.90	48.14	47.69	22.48 ± 0.26
S10	3.49	33.15	32.05	14.84 ± 0.03
S11	2.57	8.82	7.21	2.28 ± 0.10
S12	1.54	3.31	1.10	1.85 ± 0.05

Supplementary Figure S8 illustrates TG analysis curves of the solid product obtained at different reaction conditions mentioned in Table 1. The overall weight loss will occur in three different transition stages, such as removal of surface-adsorbed water (25–280 °C), elimination of chemically bound water (280–600 °C), and finally, the calcium carbonate decomposition at T > 600 °C. This decomposition phase led to the CO_2_ being released in the gas phase, resulting in weight loss in the material. According to Eq. (3), this weight loss could easily calculate the residual calcium carbonate content^24^.

3 $${\text{CaCO}}_{3}= \text{CaO}+{\text{CO}}_{2}.$$

Different weight losses were observed in the temperature range of 25–1000 °C, as seen in the TGA profile (Supplementary Fig. S8b). Upon increasing the reaction time to 2 h, the content of remaining calcium carbonate decreased from 2.66 to 1.1%. A substantial decrease in carbonate content from 17 to 1.1% was observed when the temperature increased from room temperature to 160 °C (Supplementary Fig. S8c). Supplementary Figure S8a represents the crucial effect of the stirring rate on the advancement of the reaction. At 160 °C, the decomposition of calcium carbonate reached near completion after only 2 h (~ 99%). But in the case of the S1 and S9 samples, the amount of unreacted calcium carbonate was 17.49% and 47.69%, respectively, thus indicating incomplete transformation of calcium carbonate into HAp. The TG analysis confirmed that the process parameters outlined in this study are essential for converting calcium carbonate (calcite) into HAp. The samples synthesized at the optimal parameters of 160 °C, 2 h, and 300 rpm were selected for further characterization.

### Particle size analysis and BET surface analysis

A laser diffraction-based particle size analyzer evaluated the particle size and its distribution. The results shown in Supplementary Fig. S4b,b1 confirm that the precursor calcite has a median diameter (D50) of 17.35 ± 1.6 µm, which is comparatively lesser than the HAp (18.37 ± 1.4 μm). The size difference between precursor and final HAp is due to the dissolution and precipitation reaction. We have also calculated the size distribution through SEM images using ImageJ software and reported the histograms in Supplementary Figs. S5–S7. The histogram profiles supported the particle size analysis trend. Still, the values of the precursor and resultant HAp reported in the particle size analysis method differ from those obtained from a morphological evaluation using SEM images. This variation is evident because the particle size analyzer will give hydrodynamic size, but SEM analysis will provide dry powder size.

Supplementary Figure S4a,a1 shows the N_2_ adsorption and desorption profile of the S3 sample and its precursor. The calculated surface area found from this is 15 m^2^/g with the respective pore sizes of 32 nm. The HAp shows a type IV isotherm with an H3 hysteresis loop (P/P0 > 0.4), representing the existence of non-rigid clusters of plate-like microspheres forming a slit-shaped mesoporous pore. The precursor calcite shows Type III isotherm with 0.92 m^2^/g surface area and 12.15 nm pore size. Even though the precursor material has less surface area, the HAp formed showed better surface area due to the dissolution precipitation process during the formation process^25^.

The current synthesis method using calcite and diammonium phosphate uses less energy by avoiding the hydration and calcination steps and is therefore advantageous to the existing industrial process using orthophosphoric acid and calcium hydroxide which use these steps^9^.

### Separation of binary protein mixtures

**Figure 8:**
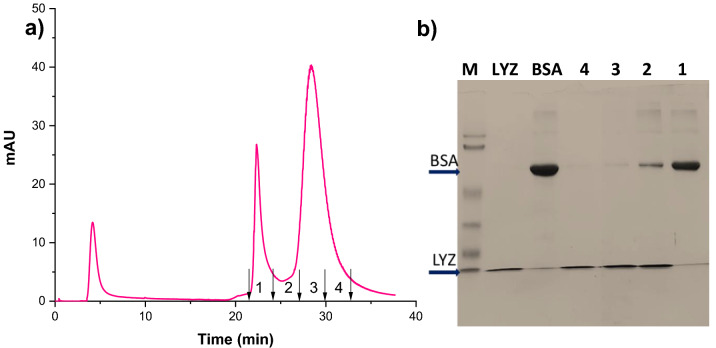
Isocratic elution chromatogram for the binary protein mixture of BSA and LYZ (**a**), SDS-PAGE cropped image obtained for collected fractions with marker and standard proteins (**b**).

Figure 8a shows the isocratic elution chromatograms for the binary mixture of BSA and LYZ at a flow rate of 1 ml/min. The chromatogram results proved that the prepared HAp microspheres could separate protein mixtures into individual proteins. The separation parameters indicated in Table 3 confirmed that the two peaks are distinct since the selectivity and resolution factor showed higher values. The BSA protein is eluted first, followed by lysozyme, which is confirmed by SDS-Polyacrylamide gel electrophoresis (SDS-PAGE) analysis. The fractions collected from the experiments were analyzed using 12% SDS-PAGE under reducing conditions. The gel was stained with Coomassie brilliant blue dye for better protein band visualization, and the resultant image is shown in Fig. 8b. The BSA protein is eluted first without any LYZ impurity, and the intermediate fractions contain both the proteins, but the last fraction contains only LYZ.

**Table 3:** Column separation parameters of HAp matrix.

Sample	Selectivity factor	Plates/meter	Resolution
HAp	1.34	4481	3.52

## Conclusion

A single-step approach has been developed to utilize calcite as a precursor for HAp microsphere preparation. The role of different physical parameters in converting calcium carbonate (calcite) to hydroxyapatite (HAp) was studied. The XRD results suggest that the existence of a prominent calcite peak at 29.6° (104) confirms the incomplete conversion of the process, which is more evident in the lower value of all the process conditions. The FTIR and TG analysis proved that the stirring conditions, reaction time, and temperatures are the significant parameters that need to be optimized. The samples prepared under optimized conditions show a negligible amount of carbonate presence, confirming the complete conversion process for the formation of microsphere HAp. The results were further supported by SEM and EDS spectra. The chromatography column prepared with the microsphere HAp demonstrated good protein separation efficiency for a binary protein mixture. The HAp microspheres obtained from calcite may be an exciting material for biomedical and chromatography applications due to their excellent physical properties like uniform morphology, narrow size distribution, better surface area, and high purity. In the future, further surface modifications can be implemented on HAp to improve protein separations.

## Supplementary Information

Supplementary Information.

## Data Availability

All data generated or analysed during this study are included in this published article [and its Supplementary Information files]. Any further details on data generated and/or analysed during the current study are available from the corresponding author on reasonable request.

## References

[1] Pham Minh D, Tran ND, Nzihou A, Sharrock P (2013). One-step synthesis of calcium hydroxyapatite from calcium carbonate and orthophosphoric acid under moderate conditions. Ind. Eng. Chem. Res..

[2] PhamMinh D, Lyczko N, Sebei H, Nzihou A, Sharrock P (2012). Synthesis of calcium hydroxyapatite from calcium carbonate and different orthophosphate sources: A comparative study. Mater. Sci. Eng. B Solid State Mater. Adv. Technol..

[3] Huang H, Du M, Chen J, Zhong S, Wang J (2020). Preparation and characterization of abalone shells derived biological mesoporous hydroxyapatite microspheres for drug delivery. Mater. Sci. Eng. C.

[4] Ashokan A, Rajendran V, Kumar TSS, Jayaraman G (2021). Eggshell derived hydroxyapatite microspheres for chromatographic applications by a novel dissolution-precipitation method. Ceram. Int..

[5] Long T, Guo Y-P, Liu Y-Z, Zhu Z-A (2013). Hierarchically nanostructured mesoporous carbonated hydroxyapatite microspheres for drug delivery systems with high drug-loading capacity. RSC Adv..

[6] Bohner M (2013). Synthesis of spherical calcium phosphate particles for dental and orthopedic applications. Biomatter.

[7] Pham Minh D (2014). Hydroxyapatite starting from calcium carbonate and orthophosphoric acid: Synthesis, characterization, and applications. J. Mater. Sci..

[8] Gecim G, Dönmez S, Erkoc E (2021). Calcium deficient hydroxyapatite by precipitation: Continuous process by vortex reactor and semi-batch synthesis. Ceram. Int..

[9] Shavandi A, Bekhit AEDA, Ali A, Sun Z (2015). Synthesis of nano-hydroxyapatite (nHA) from waste mussel shells using a rapid microwave method. Mater. Chem. Phys..

[10] Baláž M (2014). Eggshell membrane biomaterial as a platform for applications in materials science. Acta Biomater..

[11] Oliveira DA, Benelli P, Amante ER (2013). A literature review on adding value to solid residues: Egg shells. J. Clean. Prod..

[12] Xia X (2018). Synthesis of hollow structural hydroxyapatite with different morphologies using calcium carbonate as hard template. Adv. Powder Technol..

[13] Lin K, Wu C, Chang J (2014). Advances in synthesis of calcium phosphate crystals with controlled size and shape. Acta Biomater..

[14] Anbu P, Kang CH, Shin YJ, So JS (2016). Formations of calcium carbonate minerals by bacteria and its multiple applications. Springerplus.

[15] Wang Y, Moo YX, Chen C, Gunawan P, Xu R (2010). Fast precipitation of uniform CaCO3 nanospheres and their transformation to hollow hydroxyapatite nanospheres. J. Colloid Interface Sci..

[16] He Z, Xia Z, Zhang M, Wu J, Wen W (2019). Calcium carbonate mineralization in a surface-tension-confined droplets array. Crystals.

[17] Wang M (2019). Immobilization of cadmium by hydroxyapatite converted from microbial precipitated calcite. J. Hazard. Mater..

[18] Yoshimura M (2004). Hydrothermal conversion of calcite crystals to hydroxyapatite. Mater. Sci. Eng. C.

[19] Verwilghen C (2009). Convenient conversion of calcium carbonate to hydroxyapatite at ambient pressure. Mater. Sci. Eng. C.

[20] Yong I, Koichi K, Ohtsuki C (2014). Hydroxyapatite formation through dissolution—precipitation reaction: Effects of solubility of starting materials. Ceram. Int..

[21] Landi E, Celotti G, Logroscino G, Tampieri A (2003). Carbonated hydroxyapatite as bone substitute. J. Eur. Ceram. Soc..

[22] Grunenwald A (2014). Revisiting carbonate quantification in apatite (bio)minerals: A validated FTIR methodology. J. Archaeol. Sci..

[23] Verma AH (2019). Curcumin releasing eggshell derived carbonated apatite nanocarriers for combined anti-cancer, anti-inflammatory and bone regenerative therapy. J. Nanosci. Nanotechnol..

[24] Yanyan S (2020). Effects of amino acids on conversion of calcium carbonate to hydroxyapatite. RSC Adv..

[25] Guo XH, Wang WN (2014). Facile fabrication of porous hydroxyapatite monoliths: Their enhanced bioactivity and adsorption capability for heavy metal ions. J. Nanomed. Nanotechnol..

