# Supplementary material for: Process optimization for the rapid conversion of calcite into hydroxyapatite microspheres for chromatographic applications
Source: Sci Rep. 2022 Jul 16;12:12164. doi: 10.1038/s41598-022-16579-4 (PMC9288468; doi:10.1038/s41598-022-16579-4)
Supplement: Supplementary file 1 — Supplementary Information. [file 41598_2022_16579_MOESM1_ESM.pdf]

Process optimization for the rapid conversion of calcite into hydroxyapatite microsphere for chromatography application

Anbuthangam Ashokan [1, 2], T S Sampath Kumar [1*], and Guhan Jayaraman [2*]

[1] Department of Metallurgical and Materials Engineering, Indian Institute of Technology Madras, Chennai-600036

[2] Department of Biotechnology, Indian Institute of Technology Madras, Chennai-600036

E-mail: anbuthangamatg@gmail.com, guhanj@iitm.ac.in *, tssk@iitm.ac.in *

Corresponding authors: Dr. T S Sampath Kumar (tssk@iitm.ac.in), Dr. Guhan Jayaraman (guhanj@iitm.ac.in)

Crystallinity and crystal size calculation

The crystallinity was measured using the below equation.

$$X_C = 100 \times \frac{I_{300} - I_{112/300}}{I_{300}}$$

$I_{112/300}$ is the intensity of the neck between (112) and (300) diffraction peaks, and I_{300} is the intensity of (300) diffraction peaks of HAp.

The crystal size was measured using the Scherrer equation.

$$D = \frac{K\lambda}{\beta \cos \theta}$$

Where D is the crystal size; K is the shape factor (0.9); λ is the wavelength of CuKα radiation in nanometer (λ = 0.15405 nm); θ is the diffracted angle of the peak; β is the full width at half maximum of the peak in radians.

Table S1. Average crystal size and crystallinity of different samples

**Samples**	**Crystallite size (nm)**	**Crystallinity (%)**
**S1**	50.93	85.94
**S2**	45.28	76.39
**S3**	45.29	76.39
**S4**	45.28	76.39

**S5**	40.75	68.75
**S6**	54.34	91.67
**S7**	50.94	85.94
**S8**	45.27	76.39
**S9**	-	-
**S10**	-	-
**S11**	32.60	55.00
**S12**	45.27	76.39

The S9 and S10 samples do not show characteristics peak of HAp.

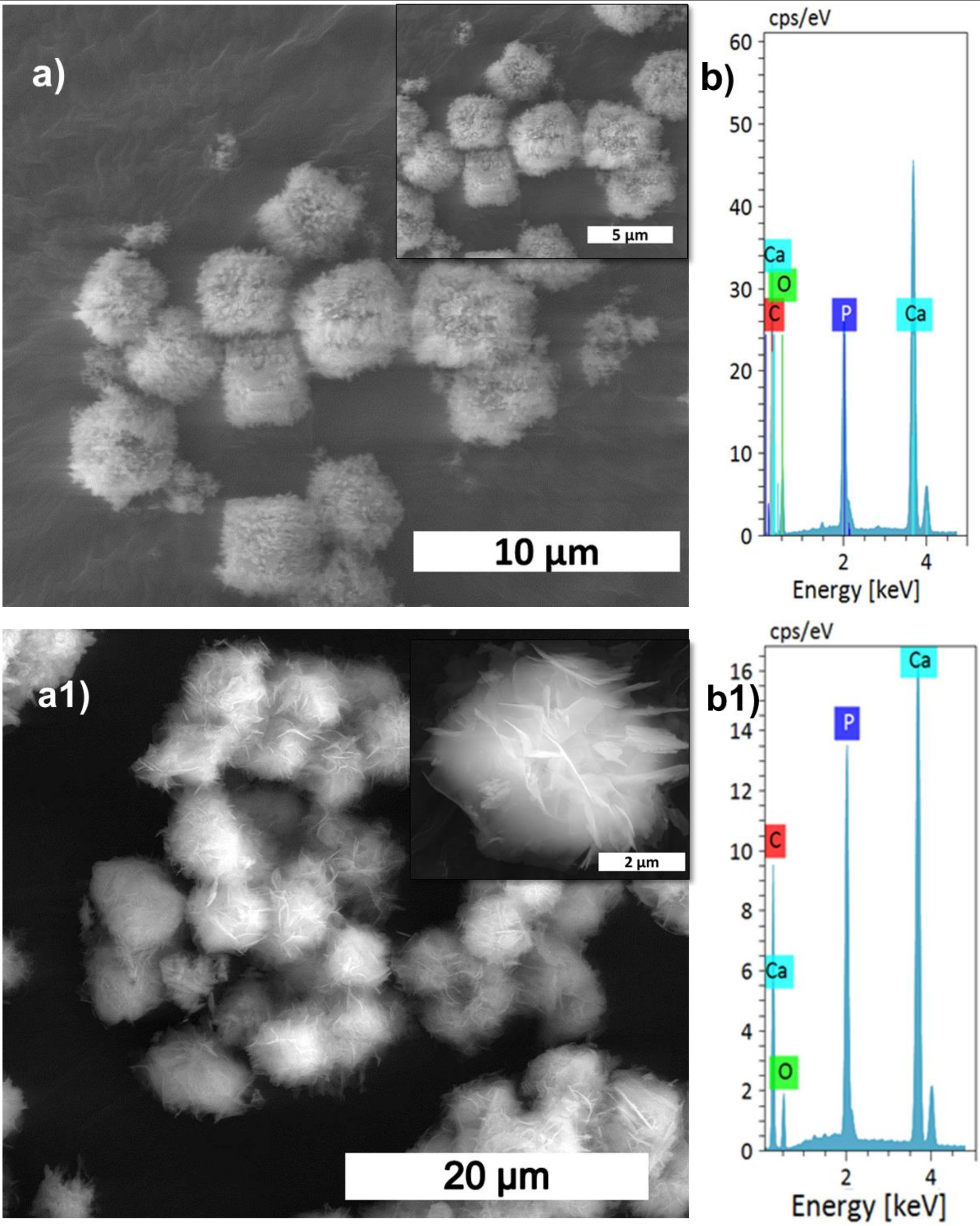

Figure S1. SEM images (a,a1) and its magnified view(insert) and EDS analysis (b,b1) of S2, S3 samples respectively.

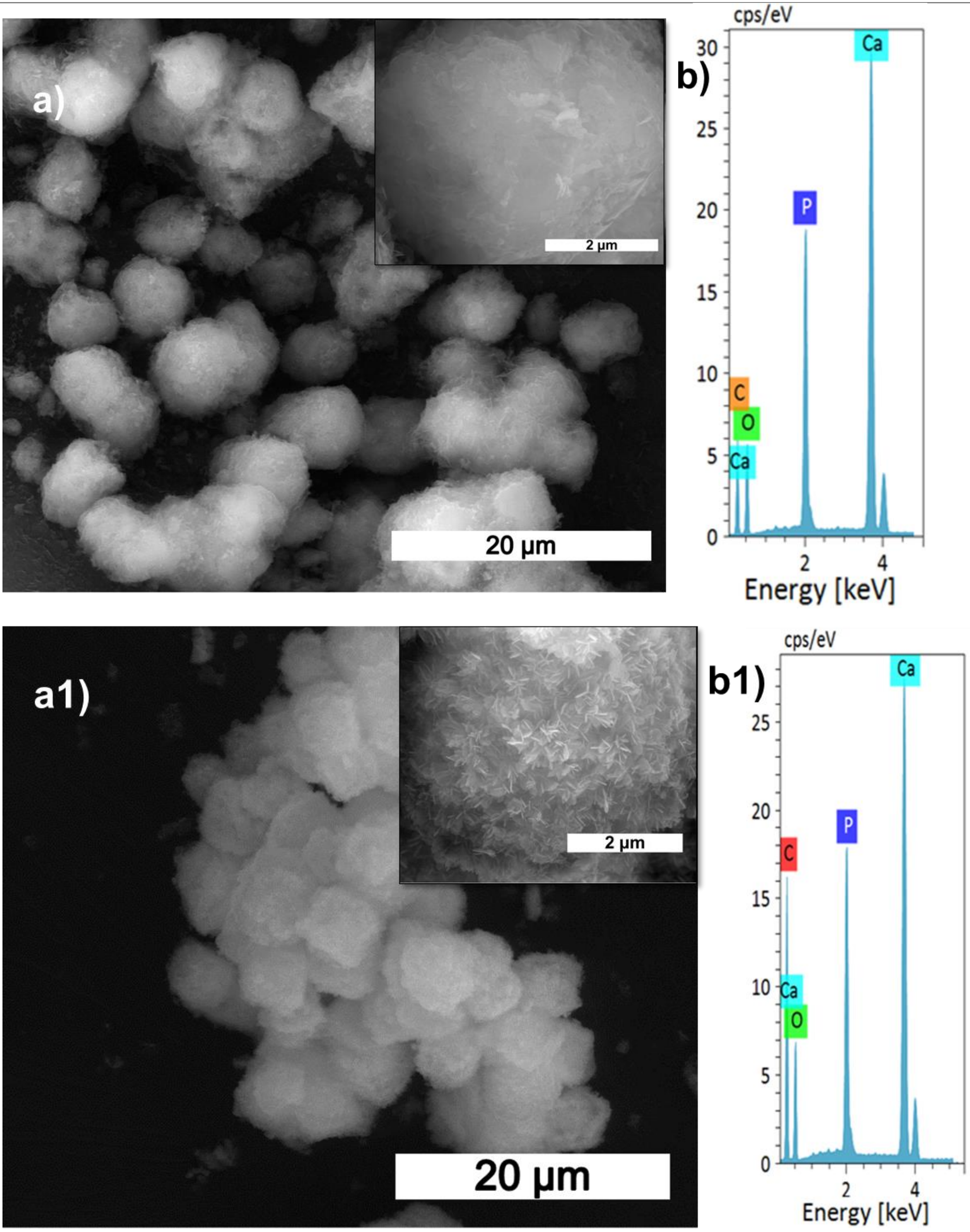

Figure S2. SEM images (a,a1) and its magnified view(insert) and EDS analysis (b,b1) of S6, S7 samples respectively.

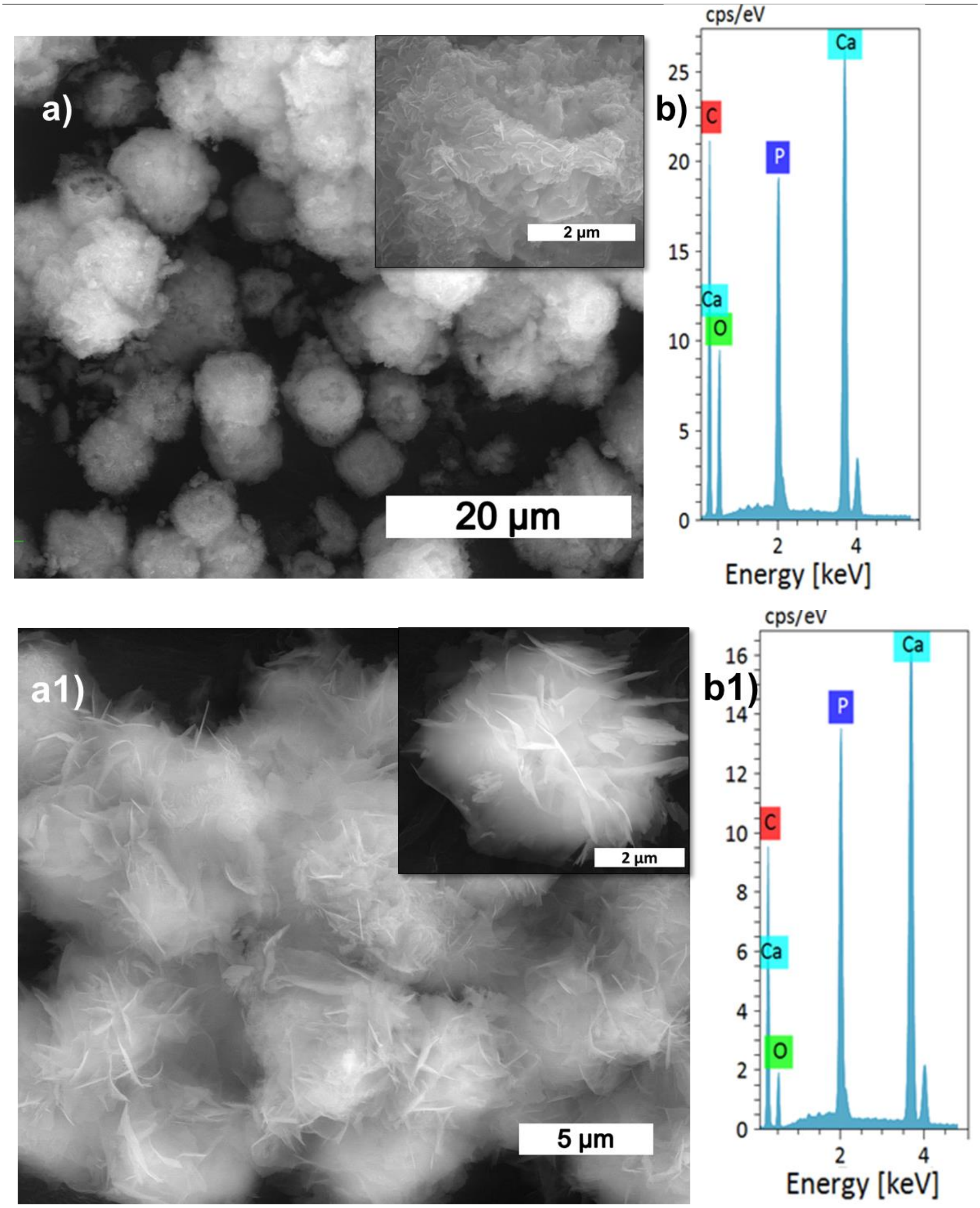

Figure S3. SEM images (a,a1) and its magnified view(insert) and EDS analysis (b,b1) of S11, S12 samples respectively.

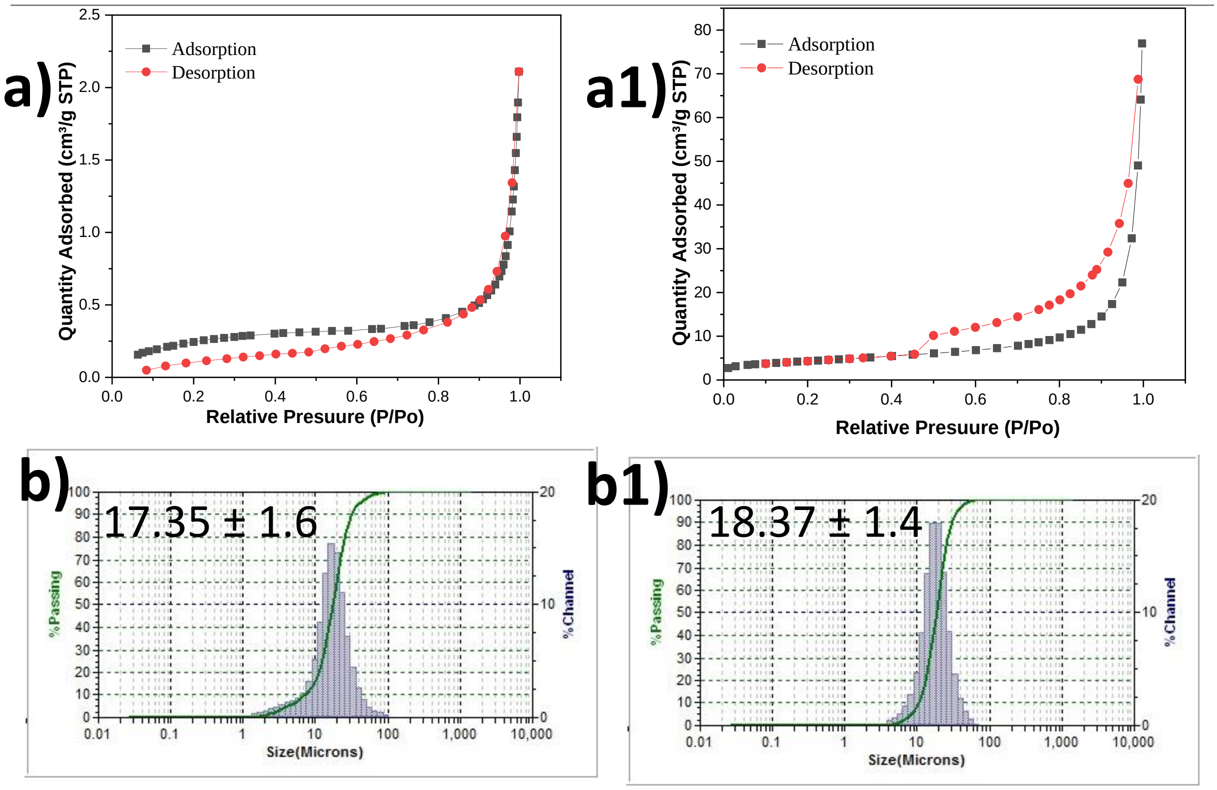

Figure S4. Nitrogen adsorption-desorption isotherm (a, a1) and particle size distribution of (b, b1) calcite and HAp, respectively.

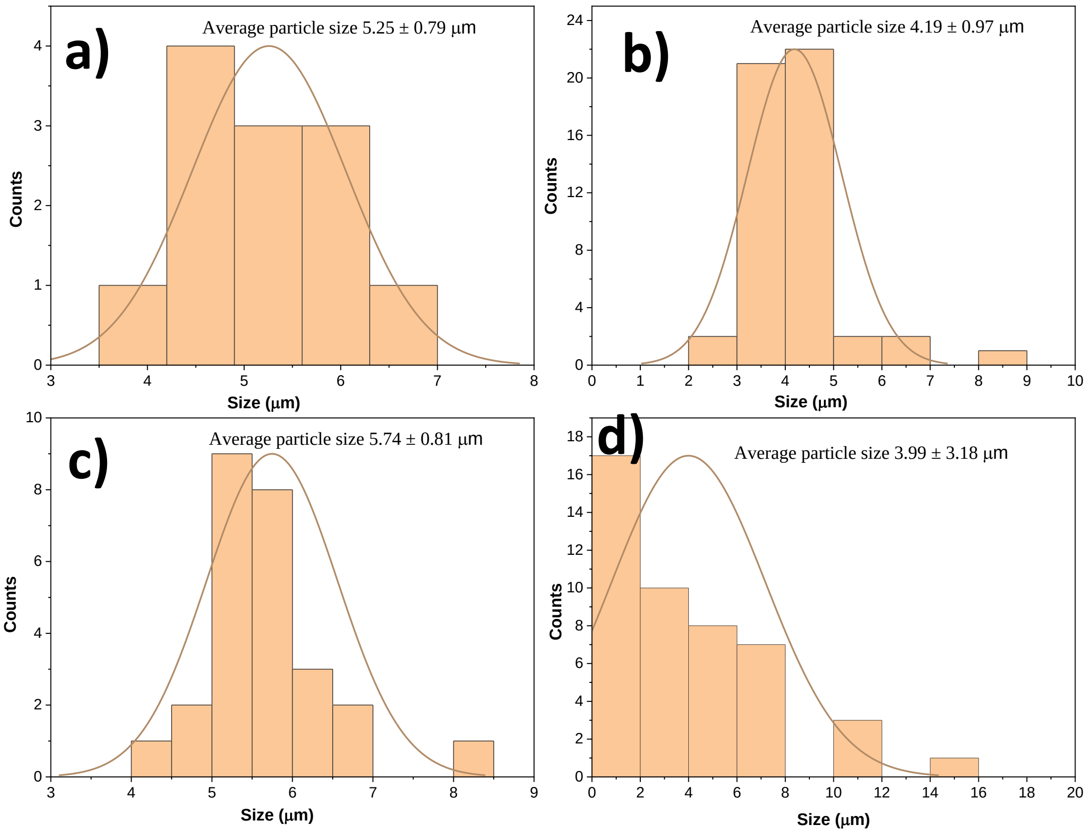

Figure S5. SEM-derived particle size distribution histogram (a-d) of S1-S4 samples respectively.

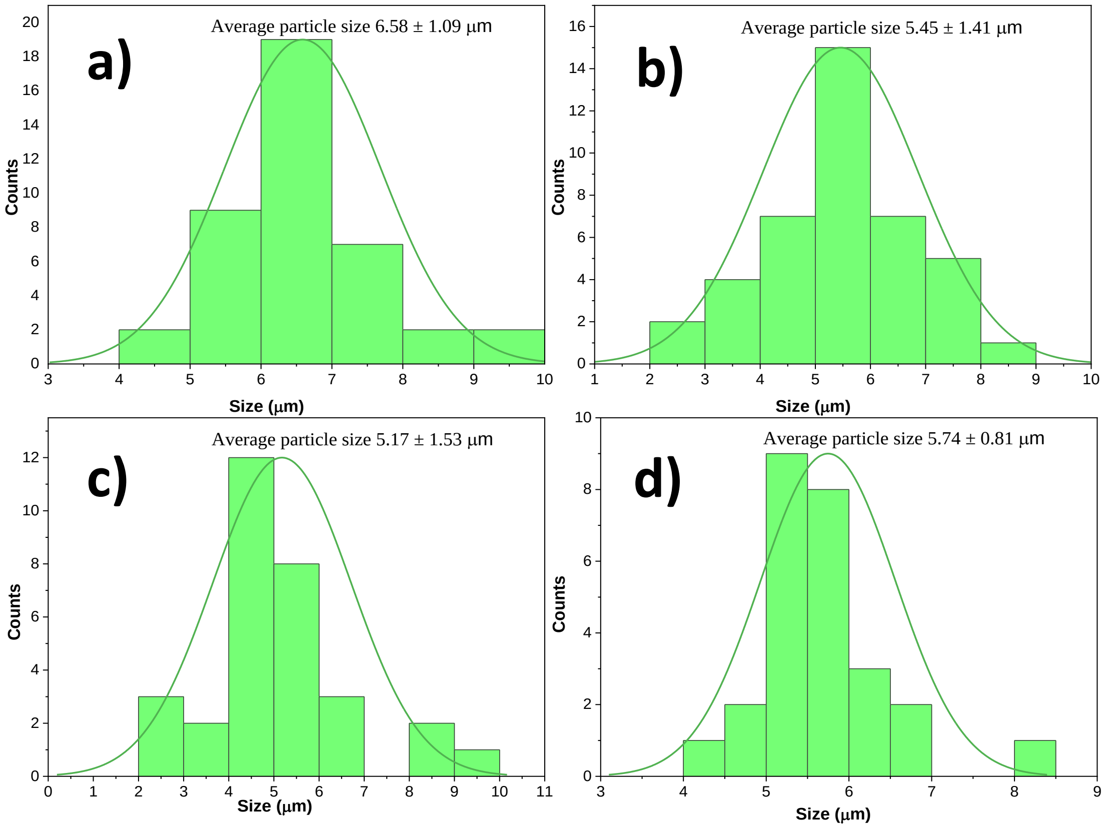

Figure S6. SEM-derived particle size distribution histogram (a-d) of S5-S8 samples respectively.

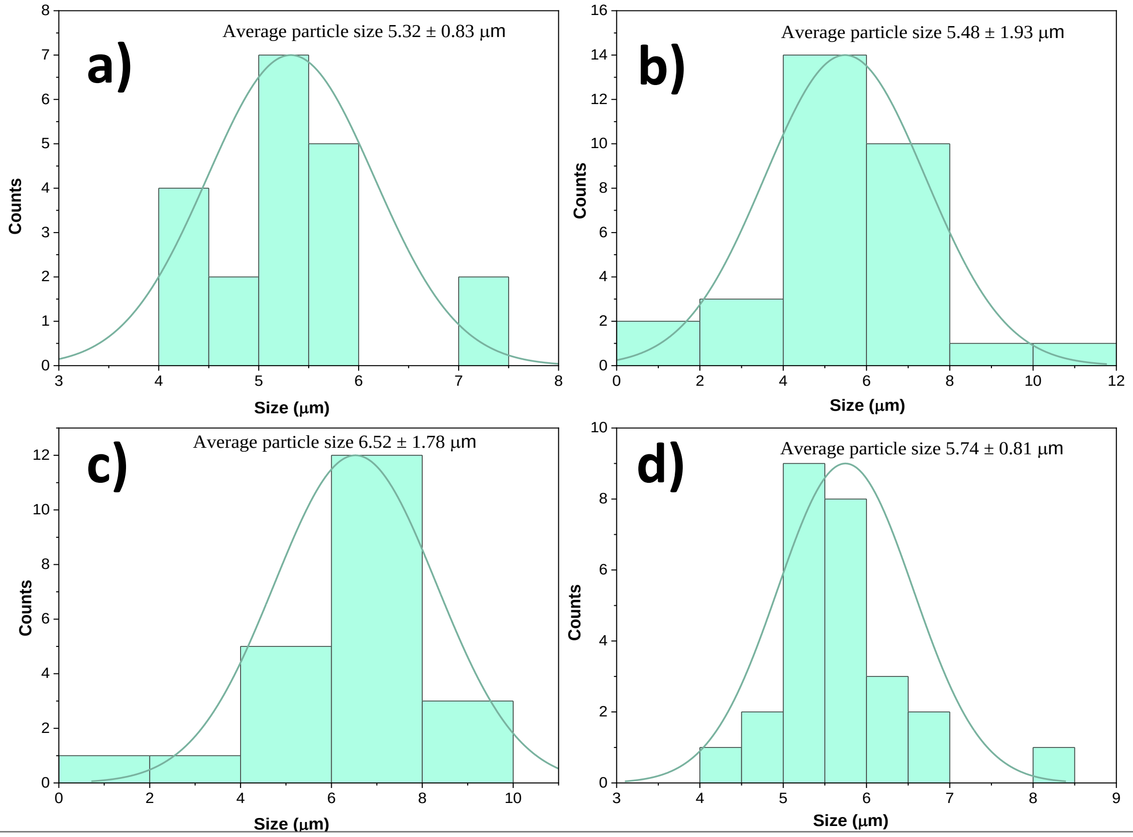

Figure S7. SEM-derived particle size distribution histogram (a-d) of S9-S12 samples respectively.

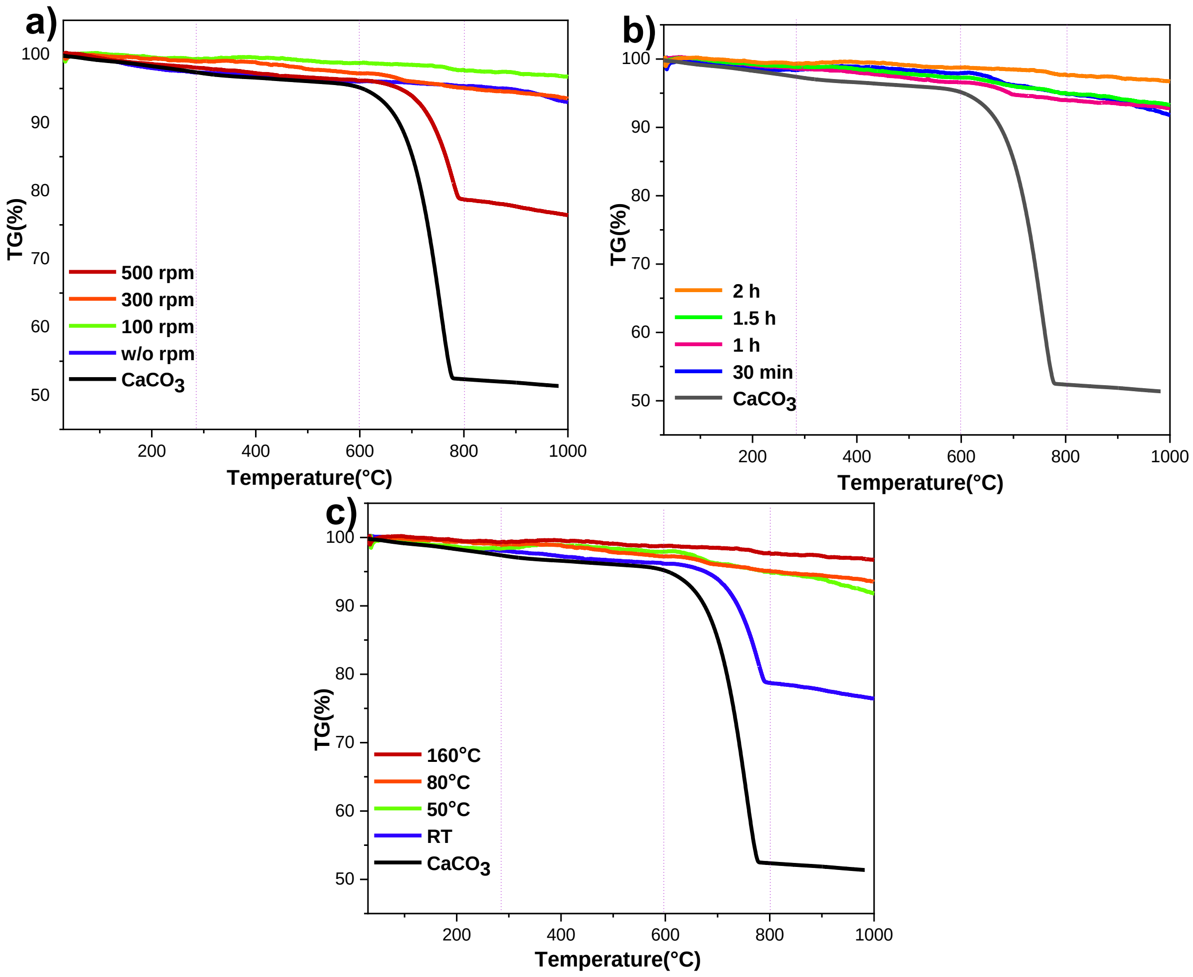

Figure S8. TG analysis of HAp samples prepared at a different stirring rate (a), time (b), and temperature (c), respectively.

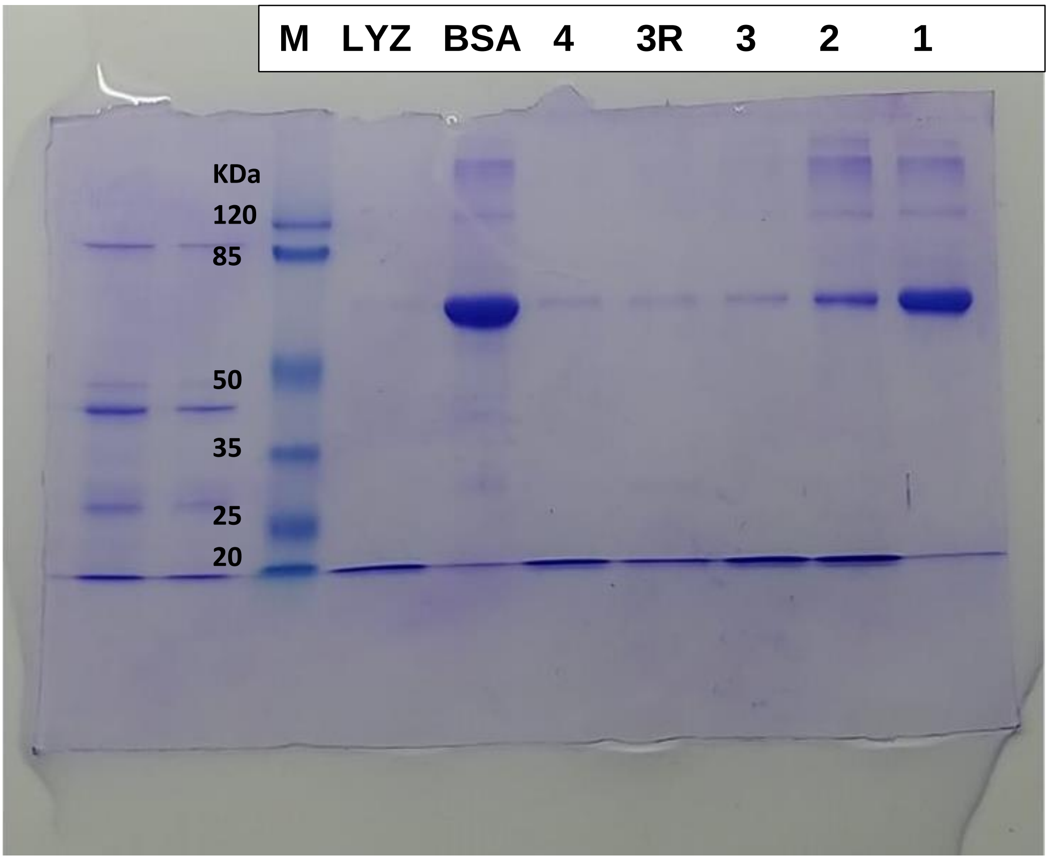

Figure S9. SDS-PAGE original image obtained for collected fractions with marker and standard proteins. Fraction 3 was repeated in SDS-PAGE and marked as 3R.Note: The first two wells are not part of the current work from the left.
